# Correlations Between County-Level Social Determinants of Health and Traumatic Brain Injury-Related Mortality in the United States

**DOI:** 10.1089/neur.2024.0100

**Published:** 2025-01-20

**Authors:** Noor Shaik, Connor A. Law, Alexa E. Walter, Eric Stulberg, Andrea L.C. Schneider

**Affiliations:** ^1^ Department of Neurology, University of Pennsylvania Perelman School of Medicine, Philadelphia, USA.; ^2^ Department of Neurology, University of Utah Spencer Fox Eccles School of Medicine, Philadelphia, USA.; ^3^ Department of Biostatistics, Epidemiology, and Informatics, University of Pennsylvania Perelman School of Medicine, Philadelphia, USA.

**Keywords:** TBI, Social Determinants of Health, TBI-Related Mortality

## Abstract

Nationally representative associations of social determinants of health (SDoH) and health care access metrics with TBI-related mortality are not well described and may differ by age. Using the Centers for Disease Control and Prevention Wide-ranging Online Data for Epidemiological Research platform and other publicly available datasets, we investigated correlations between county-level measures of SDoH (multidimensional deprivation index, social deprivation index, rural–urban continuum codes) and health care access (median distance to nearest emergency department, trauma center, intensive care unit [ICU], number of hospitals and number of hospitals with ICU capability per 1,000 population) with county-level TBI-related mortality overall and stratified by age in the United States from 1999 through 2020. Data from 2,970 counties (95.4% of eligible U.S. counties) were included. We observed a modest correlation of higher county-level TBI-related mortality with greater rurality (ρ = 0.54, 95% CI = 0.52–0.57, *R*^2^ = 0.30). Higher county-level TBI-related mortality was also modestly correlated with farther county-level median distance to nearest hospital with ICU capability (ρ = 0.43, 95% CI = 0.39–0.46, *R*^2^ = 0.18). Correlations with SDoH and health care access measures were stronger for county-level TBI-related mortality among younger (aged <50 years) compared to among older (aged ≥75 years) individuals. In conclusion, rurality and access to hospitals with ICU level care are correlated with county-level TBI-related mortality, with rurality accounting for 30% of the observed variance in county-level TBI-related mortality. Rural communities with limited access to ICUs should be targeted for prevention efforts of TBI-related deaths among younger individuals, while additional work is needed to determine factors related to variation in TBI-related mortality among older individuals.

## Introduction

Mortality associated with traumatic brain injury (TBI) increased between 2010 and 2020, particularly among older adults.^1^ It is increasingly recognized that differences in social determinants of health (SDoH) and access to health care impact both morbidity and mortality.^2,3^ SDoH broadly encompasses sociocultural, environmental, and structural factors that contribute to health outcomes and mortality via both direct and indirect mechanisms (e.g., pro-inflammatory pathways).^4^ Specifically, SDoH include differences in access to quality education, occupational opportunities, environmental exposures, exposure to discrimination/systemic racism, and food and housing security, among other factors.^5^ Several prior studies have investigated associations of SDoH and health care access with TBI-related mortality, showing associations of greater rurality^6–8^ and higher social vulnerability (defined by factors related to income/poverty, unemployment, lower education, and crowded housing, among others) with worse outcomes after TBI.^9,10^ These prior studies have limitations in generalizability with regards to geography (i.e., were not nationally representative)^7^ and age (i.e., did not evaluate across the lifespan).^8–10^ Prior studies have also not comprehensively compared which measures of SDoH and health care access are most strongly related to TBI-mortality.^2,3^ Nationally representative examinations of relationships of SDoH and health care access with TBI-related mortality across the lifespan are needed to identify vulnerable populations and to help focus efforts to reduce TBI-related mortality. We aimed to investigate correlations between county-level measures of SDoH and health care access with county-level TBI-related mortality in the United States from 1999 to 2020, both overall and stratified by age.

## Materials and Methods

### County-level TBI-related mortality data

Data on county-level TBI-related mortality rates per 100,000 individuals were obtained from the National Vitals Statistics System multiple-cause-of-death files available via the Centers for Disease Control and Prevention (CDC) Wide-ranging Online Data for Epidemiological Research (WONDER) platform for 1999–2020.^11^ We obtained county-level TBI-related mortality data overall and stratified by age (<25 years, 25-<50 years, 50-<75 years; ≥75 years). TBI-related mortality was defined in accordance with the CDC surveillance definition (injury-related International Classification of Disease, Tenth Revision [ICD-10] underlying cause of death code [V01-Y36, Y85-Y87, Y89, U01-U03] and a TBI-related ICD-10 code in at least one of the 20 multiple-cause-of-death fields [S01, S02.0, S02.1, S02.3, S02.7–S02.9, S04.0, S06, S07.0, S07.1, S07.8, S07.9, S09.7–S09.9, T90.1, T90.2, T90.4, T90.5, T90.8, T90.9]).^12^ We excluded counties which had changes in the borders over time (all counties in Alaska and Bedford County, Virginia and Broomfield County, Colorado) and counties with <20 total TBI-related mortality events for 1999–2020 in accordance with CDC WONDER analysis recommendations.^13^ These exclusions resulted in 143 counties excluded from the analysis examining all ages, and the following number of counties excluded from age-stratified analyses: 1,209 for age <25 years, 753 for age 25-<50 years, 738 for age 50-<75 years, and 1,052 for age ≥75 years.

### County-level SDoH and access to health care data

County-level SDoH data included: the 2010–2019 multidimensional deprivation index (MDI)^14^ and the 2015–2019 social deprivation index (SDI).^15^ The MDI is derived from American Community Survey, County Health Rankings and Roadmaps, and U.S. Census Bureau data, and is comprised of the following six dimensions: standard of living, education, health, economic security, housing quality, and neighborhood quality.^14^ Higher MDI values represent greater deprivation. The SDI is derived from the American Community Survey and is comprised of the following components, which are all percentages of the county population: living below 100% of the federal poverty level, adults aged ≥25 years with <12 years of education, single-parent households with dependents aged <18 years, living in rented housing units, living in overcrowded housing units (defined as ≥1.01 occupants per room), households without a vehicle, and non-employed individuals aged 16–64 years.^15^ Higher SDI values represent greater deprivation. We additionally evaluated each individual component of the SDI to better understand which specific, potentially intervenable deprivation characteristics of a community may account for variation in TBI-related mortality.

County-level access to health care data included the 2023 Rural–Urban Continuum Codes^16^ and metrics drawn from the Agency for Health Care Research and Quality’s 2020 Social Determinants of Health database, including: median distance to nearest emergency department (ED), medical-surgical intensive care unit (ICU), and trauma unit, the number of hospitals per 1,000 population, and the number of hospitals with ICU capability per 1,000 population.^17^ Rural–urban continuum codes are determined by the Office of Management and Budget, which assigns counties an ordinal score of 1–9 for each county where higher values represent greater rurality such that scores of 1–3 are metropolitan, whereas 4–9 are non-metropolitan.^16^

### Statistical analysis

Spearman correlation coefficients (ρ), 95% confidence intervals (95% CIs), and *R*^2^ values were calculated between each county-level SDoH and access to health care measure and county-level TBI-related mortality rates and overall and stratified by age (<25 years, 25-<50 years, 50-<75 years; ≥75 years). We used scatter plots with locally estimated scatterplot smoothing curves (95% CIs), a technique agnostic to any particular functional form assumptions, to graphically depict overall associations between county-level TBI-related mortality and MDI, SDI, rural–urban continuum codes, median distance to nearest ED, ICU, and trauma unit, and the number of hospitals and the number of hospitals with ICU capability per 1,000 people. All statistical analyses were performed in R version 4.2.2.

Due to the deidentified nature of the data, this research is considered nonhuman research under U.S. regulation (45 CFR §46.102[d]) and did not require informed consent.

## Results

Data from 2,970 U.S. counties (95.4% of 3,113 total eligible U.S. counties) were included in the overall analyses. County-level TBI-related mortality was weakly correlated with the MDI (ρ = 0.28, 95% CI = 0.25, 0.31, *R*^2^ = 0.08) and not correlated with the composite SDI. The SDI component with the strongest, but still modest, correlation was percentage of non-employed individuals aged 16–64 years (ρ = 0.32, 95% CI = 0.29, 0.35, *R*^2^ = 0.10). Correlations with SDoH measures were stronger for county-level TBI-related mortality among younger (aged <50 years) compared to older (aged ≥75 years) individuals (Table 1).

**Table 1.** Spearman Correlations (ρ), 95% Confidence Intervals, and *R*^2^ Values Between County-Level TBI-Related Mortality and County-Level SDoH and Access to Health Care Metrics in the United States

	County-level TBI-related mortality									
	Overall		Ages 0–<25 years		Ages 25–<50 years		Ages 50–<75 years		Ages ≥75 years	
	ρ (95% CI)	*R* ^2^	ρ (95% CI)	*R* ^2^	ρ (95% CI)	*R* ^2^	ρ (95% CI)	*R* ^2^	ρ (95% CI)	*R* ^2^
County-level SDoH metrics										
Multidimensional deprivation index	0.28 (0.25, 0.31)	0.08	0.43 (0.39, 0.47)	0.18	0.49 (0.45, 0.52)	0.24	0.40 (0.36, 0.43)	0.16	−0.12 (−0.16, −0.07)	0.01
Social deprivation index	0.18 (0.14, 0.21)	0.03	0.34 (0.30, 0.38)	0.11	0.39 (0.35, 0.42)	0.15	0.33 (0.29, 0.37)	0.11	−0.15 (−0.19, −0.11)	0.02
% Living below 100% of the federal poverty level	0.29 (0.25, 0.32)	0.08	0.41 (0.37, 0.45)	0.17	0.49 (0.45, 0.52)	0.24	0.41 (0.37, 0.44)	0.17	−0.09 (−0.13, −0.04)	0.01
% of adults aged ≥25 years with <12 years of education	0.28 (0.25, 0.31)	0.08	0.48 (0.44, 0.52)	0.23	0.48 (0.44, 0.51)	0.23	0.40 (0.36, 0.43)	0.16	−0.12 (−0.16, −0.08)	0.01
% of single-parent households with dependents aged <18 Years	−0.13 (−0.16, −0.09)	0.02	0.06 (0.02, 0.11)	0.00	0.01 (−0.03, 0.05)	0.00	−0.02 (−0.06, 0.02)	0.00	−0.16 (−0.20, −0.12)	0.03
% Living in rented housing units	−0.23 (−0.26, −0.19)	0.05	−0.22 (−0.27, −0.18)	0.05	−0.18 (−0.22, −0.14)	0.03	−0.10 (−0.14, −0.06)	0.01	−0.11 (−0.15, −0.07)	0.01
% Living in overcrowded housing units	0.03 (−0.01, 0.06)	0.00	0.14 (0.10, 0.19)	0.02	0.14 (0.10, 0.18)	0.02	0.17 (0.13, 0.21)	0.03	−0.07 (−0.11, −0.02)	0.01
% of households without a vehicle	−0.04 (−0.07, 0.00)	0.00	0.03 (−0.02, 0.07)	0.00	0.05 (0.01, 0.09)	0.00	−0.01 (−0.05, 0.03)	0.00	−0.21 (−0.25, −0.17)	0.04
% Non-employed individuals aged 16–64 years	0.32 (0.29, 0.35)	0.10	0.48 (0.44, 0.52)	0.23	0.52 (0.49, 0.55)	0.27	0.45 (0.42, 0.48)	0.20	−0.07 (−0.12, −0.03)	0.01
County-level access to healthcare metrics										
Rural–urban continuum codes	0.54 (0.52, 0.57)	0.30	0.54 (0.50, 0.57)	0.29	0.53 (0.49, 0.56)	0.28	0.47 (0.44, 0.50)	0.22	0.19 (0.15, 0.24)	0.04
Median distance (miles) to nearest ED	0.27 (0.24, 0.31)	0.08	0.34 (0.30, 0.38)	0.12	0.31 (0.28, 0.35)	0.10	0.24 (0.20, 0.28)	0.06	0.11 (0.07, 0.15)	0.01
Median distance (miles) to nearest ICU	0.43 (0.39, 0.46)	0.18	0.45 (0.42, 0.49)	0.21	0.44 (0.41, 0.47)	0.19	0.35 (0.32, 0.39)	0.13	0.14 (0.10, 0.18)	0.02
Median distance (miles) to trauma center	0.25 (0.21, 0.28)	0.06	0.30 (0.26, 0.34)	0.09	0.28 (0.24, 0.32)	0.08	0.23 (0.19, 0.27)	0.05	0.05 (0.01, 0.09)	0.00
Number of hospitals per 1,000 population	−0.01 (−0.05, 0.03)	0.00	0.15 (0.11, 0.20)	0.02	0.03 (−0.01, 0.07)	0.00	0.06 (0.02, 0.10)	0.00	0.00 (−0.05, 0.04)	0.00
Number of hospitals with ICU capability per 1,000 population	0.34 (0.31, 0.37)	0.12	0.44 (0.41, 0.48)	0.20	0.36 (0.33, 0.40)	0.13	0.34 (0.31, 0.38)	0.12	0.13 (0.09, 0.17)	0.02

95% CI, 95% Confidence Interval; ED, emergency department; ICU, intensive care unit; SDoH, social determinants of health.

**FIG. 1.**
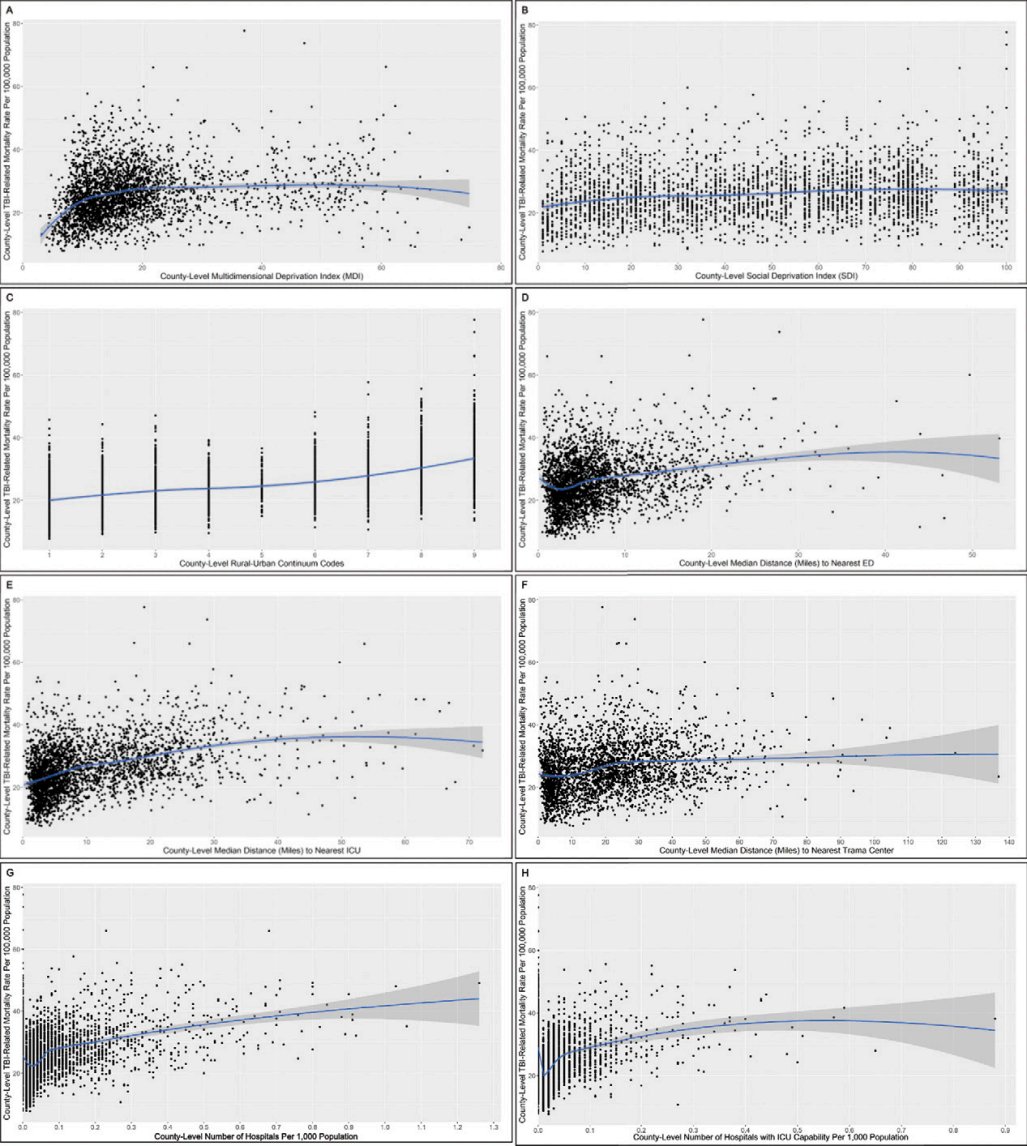
Scatterplots and locally estimated scatterplot smoothing (LOESS) curves (95% CIs) for associations between county-level TBI-related mortality and county-level social determinants of health (SDoH) and access to healthcare metrics in the United States.

We observed a modestly strong positive correlation between county-level TBI-related mortality with the rural–urban continuum codes, such that greater rurality accounted for 30% of the variation in county-level TBI-related mortality (ρ = 0.54, 95% CI = 0.52, 0.57, *R*^2^ = 0.30) (Table 1, Fig. 1). County-level TBI-related mortality was also modestly correlated with farther county-level median distance to nearest hospital with ICU capability (ρ = 0.43, 95% CI = 0.39, 0.46, *R*^2^ = 0.18). There were weak to moderate correlations of greater county-level TBI-related mortality with farther county-level median distance to nearest ED and trauma center. There was no correlation between county-level TBI-related mortality and the county-level number of hospitals per 1,000 population. Correlations with access to health care metrics were moderately positive for county-level TBI-related mortality among younger (aged <50 years) individuals and were negligible among older (aged ≥75 years) individuals (Table 1). For example, rurality accounted for 29% and 28% of the variance in county-level TBI-related mortality rates among individuals aged <25 years and 25-<50 years, respectively. In contrast, rurality only accounted for only 4% of the variance among individuals aged ≥75 years.

## Discussion

Using nationally representative U.S. county-level data, we found that composite and individual SDoH and access to health care factors at best modestly correlate with TBI-related mortality. Correlations between county-level SDoH and access to health care factors with county-level TBI-related mortality were stronger among younger compared to older individuals, with negligible correlations observed for county-level SDoH and access to health care factors with county-level TBI-related mortality among individuals aged 75 years or older.

We found that greater county-level rurality had the strongest correlation among factors examined with county-level TBI-related mortality, explaining 30% of the variance, followed by greater median distance to the nearest ICU, which explained 18% of the variance. These two county-level access to health care factors were more strongly correlated with county-level TBI-related mortality than were county-level SDoH measures. These findings suggest that with regards to TBI-related mortality, factors related to health care access may be more important than factors related to poverty, education, crowded housing units, and employment, among others. Our nationally representative county-level findings expand upon, and are consistent with, previously published state-level data showing that states with a higher proportion of residents living in rural areas had higher rates of TBI-related mortality in 2016–2018.^6^ Similarly, other county-level data from Colorado showed that both TBI-related hospitalization and TBI-related mortality were higher in rural compared to urban counties.^7^ The importance of access to health care resources is consistent with prior studies showing that geographic regions with a greater number of trauma centers had lower trauma-related mortality rates^18^ and that trauma patients treated at higher level trauma centers had better outcomes than patients treated at lower level trauma centers.^19^

In age-stratified analyses, the proportion of variance in county-level TBI-related mortality explained by greater county-level rurality and by greater median distance to nearest hospital with ICU capacity (and other SDoH and health care access metrics) was higher in individuals aged <50 years and significantly lower for individuals aged 75 years or older. Other work has reported that ICU-level care is associated with improved functional outcomes after TBI, but not with reduction in 6-month mortality rates.^20^ It has been suggested that changing demographics of patients admitted to the ICU over the past two decades (increasing median age of patients) may contribute to this discrepancy, since younger patients with severe TBI treated in the ICU tend to have better outcomes over time but older patients continue to have poor outcomes, even with milder TBI.^21,22^ In the setting of significant increases in TBI-related mortality rates among individuals aged 75 years and older over the past two decades,^1^ future research focused on identifying factors underlying the observed age-related disparities is critically needed to inform studies designed to prevent TBI and improve outcomes for older individuals. In contrast, the stronger correlations between TBI-related mortality and ICU-level care and rurality among younger individuals (aged <50 years old) suggest that rural communities with limited access to ICUs should be targeted for prevention efforts of TBI-related deaths among younger individuals.

Certain limitations should be taken into consideration. It is important to remember that our county-level study is ecological in nature, precluding extrapolation of our findings to an individual level (i.e., ecological fallacy). Further, this data does not allow for causal inference. Due to small numbers of TBI-related deaths (<20) occurring in certain counties overall and within certain county/age strata over the cumulative 22-year period of TBI-related mortality ascertainment, we were unable to include data from every U.S. county in our analyses, but our analytic sample was nationally representative with over 95% of county-level TBI-related mortality data being included in our overall analyses.

## Conclusions

In conclusion, this study demonstrates that disparities in county-level health care access, especially rurality and poor access to hospitals with ICU level care, are correlated with county-level TBI-related mortality, particularly in younger individuals. Rural communities with limited access to ICUs should be targeted for prevention efforts of TBI-related deaths among younger individuals, while future work focused on identifying other contributors should be explored to inform targeted TBI-related mortality prevention strategies among older individuals.

## Abbreviations Used

CDC: Centers for Disease Control and Prevention
CI: confidence interval
ED: Emergency department
ICD-10: International Classification of Disease, Tenth Revision
ICU: Intensive care unit
LOESS: Locally estimated scatterplot smoothing
MDI: Multidimensional deprivation index
SDI: Social deprivation index
SDoH: social determinants of health
TBI: traumatic brain injury
WONDER: Wide-ranging Online Data for Epidemiological Research
